# Corrigendum: Picture-induced semantic interference reflects lexical competition during object naming

**DOI:** 10.3389/fpsyg.2017.01002

**Published:** 2017-06-12

**Authors:** Sabrina Aristei, Pienie Zwitserlood, Rasha Abdel Rahman

**Affiliations:** ^1^Neurocognitive Psychology, Department of Psychology, Humboldt-Universität zu BerlinBerlin, Germany; ^2^Psycholinguistics and Cognitive Neuroscience Unit, Institute for Psychology, Westfälische-Wilhelms-Universität MünsterMünster, Germany

**Keywords:** speech production, picture–picture interference, lexical competition, compound naming, semantics

In the original article, the name of one of the authors was incorrectly spelled in the citation section (last page, last paragraph) as **Rahman RA**. It should be **Abdel Rahman R**. The authors apologize for this error and state that this does not change the scientific conclusions of the article in any way.

*Citation: Aristei S, Zwitserlood P and Rahman RA (2012) Picture-induced semantic interference reflects lexical competition during object naming. Front. Psychology* ***3****:28. doi: 10.3389/fpsyg.2012.00028*

*This article was submitted to Frontiers in Language Sciences, a specialty of Frontiers in Psychology*.

*Copyright*© *2012 Aristei, Zwitserlood and Rahman*.

*Citation: Aristei S, Zwitserlood P and Abdel Rahman R (2012) Picture-induced semantic interference reflects lexical competition during object naming. Front. Psychology* ***3****:28. doi: 10.3389/fpsyg.2012.00028*

*This article was submitted to Frontiers in Language Sciences, a specialty of Frontiers in Psychology*.

*Copyright* © *2012 Aristei, Zwitserlood and Abdel Rahman*.

## Conflict of interest statement

The authors declare that the research was conducted in the absence of any commercial or financial relationships that could be construed as a potential conflict of interest.

